# Heart Allograft Tolerance Induced and Maintained by Vascularized Hind-Limb Transplant in Rats

**DOI:** 10.1155/2013/483856

**Published:** 2013-03-12

**Authors:** Quan Liu, Yong Wang, Atsunori Nakao, Wensheng Zhang, Vijay Gorantla, Xin Xiao Zheng

**Affiliations:** ^1^Thomas E. Starzl Transplantation Institute, University of Pittsburgh, Pittsburgh, PA 15261, USA; ^2^Department of Plastic and Reconstructive Surgery, University of Pittsburgh, Pittsburgh, PA 15261, USA; ^3^Department of Cardiovascular Surgery, The Second Affiliated Hospital of Harbin Medical University, Harbin, Heilongjiang 150086, China; ^4^Department of Cardiothoracic Surgery, University of Pittsburgh, Pittsburgh, PA 15261, USA; ^5^Research Center for Translational Medicine, Tongji University, Shanghai East Hospital, Shanghai 200120, China

## Abstract

Organ/tissue transplantation has become an effective therapy for end-stage diseases. However, immunosuppression after transplantation may cause severe side effects. Donor-specific transplant tolerance was proposed to solve this problem. In this study, we report a novel method for inducing and maintaining heart allograft tolerance rats. First, we induced indefinite vascularized hind-limb allograft survival with a short-term antilymphocyte serum + Cyclosporine A treatment. Peripheral blood chimerism disappeared 6-7 weeks after immunosuppression was withdrawn. Then the recipients accepted secondary donor-strain skin and heart transplantation 200 days following vascularized hind-limb transplantation without any immunosuppression, but rejected third party skin allografts, a status of donor-specific tolerance. The ELISPOT results suggested a mechanism of clone deletion. These findings open new perspectives for the role of vascularized hind-limb transplant in the induction and maintenance of organ transplantation tolerance.

## 1. Introduction

Organ/tissue transplantation has become a standard and, under certain circumstances, the single effective therapy for patients with otherwise incurable diseases, such as renal or heart failure. Due to the severe side effects caused by life-long nonspecific immune suppression required to [1, 2] maintain the allograft function, such as nephrotoxicity, infection, and tumor, donor-specific transplant tolerance was proposed to obviate these problems.

After Owen discovered the phenomenon that fraternal bovine twins who share the same placenta are born with and tolerant to erythrocyte from each other [3], Billingham and coworkers provided evidence for the feasibility of “actively acquired tolerance” with the experiments in mice and chickens, demonstrating that animals inoculated with homologous cells as fetuses are tolerant to the skin graft from the same donor in adulthood [4]. The phenomenon described previously is not a feature of only animals; human blood-group chimera in a twin was also reported [5].

Based on these studies, hematopoietic chimerism, which designates the coexistence of the hematopoietic cells from both recipient and donor after bone marrow transplantation, has been studied as a means to induce transplant tolerance. At the present, transplant tolerance of certain organs or tissues has been induced successfully by the induction of hematopoietic chimerism with different recipient conditionings (myeloablation or cytoreduction) or a “megadose” bone marrow transplantation in animals or humans [6–9]. However, although full chimerism in which donor-derived hematopoietic cells completely replace the counterpart of the host [10] always leads to transplant tolerance [11], dissociation between transplant tolerance and mixed hematopoietic chimerism has been reported [12, 13]. Additionally, “split tolerance” has also been documented [14, 15]. In this type of tolerance, recipients are tolerized of a certain type of allograft while reject another type from the same donor, indicating an incomplete central tolerance of the donor pan-antigens.

Limb transplants usually include vascularised bone marrow intact with the hematopoietic microenvironment and stem cell niche. Emerging evidence indicates that sustained self-renewal of donor stem cells can enable protolerogenic mechanisms allowing successful weaning from immunosuppression under cover of short course regimens. In this study, we show that a short-term antilymphocyte serum + Cyclosporine A (ALS + CsA) treatment enabled indefinite vascularized hind-limb allograft survival, which induced secondary donor-strain skin and heart allograft tolerance.

## 2. Materials and Methods

### 2.1. Animals

Male Brown Norway (BN, RT1^n^), Lewis (LEW, RT1^l^), and August Copenhagen Irish (ACI, RT1^av1^) rats, weighing 200 to 250 g, were purchased from Harlan Laboratories and used as donors, recipients, and third-party donors, respectively. Animals were housed under pathogen-free conditions at the University of Pittsburgh Animal Facilities according to NIH guidelines.

### 2.2. Transplant Surgeries

Orthotopic hind-limb transplantation (HLT) in this study was performed on day 0 as previously described [16]. Briefly, the hind-limb of BN rats was amputated at the middle level of the femur. Removal of the LEW recipients corresponding hind-limb was performed in a similar fashion. The donor and recipient femurs were joined with a 16-gauge needle as an intramedullary rod. The femoral artery and vein were anastomosed with 10-0 nylon. Vascularized skin/muscle (part of hind-limb with the bone component removed) transplantation was performed in a similar manner. The LEW recipients, whose primary hind-limb transplant survived over 150 days, underwent a secondary full-thickness skin transplant from both BN and ACI donors. Hind-limb, vascularized skin/muscle, and skin grafts were monitored daily after surgery for signs of rejection such as edema, change of color, and necrosis of the skin. Symptoms of graft-versus-host disease (GVHD) were also followed up. Rejection of hind-limb and skin allografts was defined as the necrosis of the tissue.

Intra-abdominal heart transplantation was performed as described by Ono and Lindsey [17] 50 days following secondary skin transplant. No immunosuppressive therapy was applied following skin and heart transplantation. Function of the transplanted heart was assessed by daily palpation of graft contractions through the abdominal wall. Rejection was defined as the complete cessation of myocardial contractions, which was confirmed at laparotomy.

### 2.3. Experimental Groups

For vascularized hind-limb transplant, five groups were employed: Group 1: ALS, day −4 and day +1, 0.5 mL, i.p.; Group 2: CsA, day 0–20, 3 mg/kg, ip; Group 3: CsA, day 0–44, 3 mg/kg, ip; Group 4, ALS + 21-day CsA, and Group 5, ALS + 45-day CsA. For vascularized skin/muscle transplant (VSMT), LEW recipients were treated with ALS + 45-day CsA as Group 6.

### 2.4. ELISPOT

To analyse the direct pathway response, purified splenic T cells (enrichment columns, R&D Systems) from LEW recipients were incubated with CD3-depleted, gamma-irradiated, splenic LEW, BN, or ACI APCs (3 × 10^4^  T  cells + 2.5 × 10^5^  APCs/well) in 96-well ELISPOT plates coated with IFN-gamma antibody. ELISPOT plates were developed 36 hours later following manufacturer's instruction (BD Biosciences).

### 2.5. Flow Cytometry

To monitor peripheral multilineage chimerism, blood cells were depleted of erythrocytes and incubated with PE-CD11b/c, FITC-RT1Ac (OX-27, Serotec), APC-CD3, and PE/Cy5-CD45RA.

### 2.6. Histology

Heart allografts were harvested 150 days after transplant. Tissue was fixed in 10% buffered formalin, embedded in paraffin, sectioned, and stained with hematoxylin and eosin (H&E) using standard techniques.

### 2.7. Quantification of Donor-Derived Hematocytes by PCR Analysis

For microchimerism analysis, peripheral blood was collected from the tail vein in ethylenediaminetetraacetic-acid-(EDTA-) containing tubes. Genomic DNA was prepared with DNeasy Blood and Tissue Kit (Qiagen, California, USA) following the manufacture's protocol. Specific primers (5′-CGCAGGGGATTTCGTATT-3′ p1; 5′-GGTGGGGACCTCCGTCT-3′) were used as described previously [18].

### 2.8. Statistics

GraphPad Prism Version 5 was used for statistical analyses. Results are expressed as mean ± SD. Unpaired 2-tailed Student's *t*-test and log-rank (Mantel-Cox) test were used for the statistical analysis. A *P* value less than 0.05 was considered significant.

## 3. Results

### 3.1. ALS Is Not Sufficient for but Essential to Long-Term Vascularized Hind-Limb Transplant Acceptance in This Model

**Figure 1:**
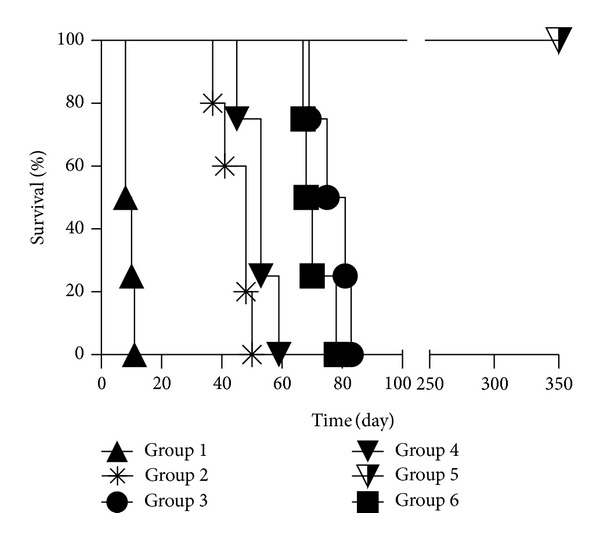
Vascularized hind-limb transplant survival time after surgery. ALS + 45-day CsA treatment induced vascularized hind-limb transplant tolerance. Other treatments failed to achieve long-term hind-limb transplant survival.

It has been well established that ALS achieves its immunosuppressive effect by bringing out a selective ablation of the population of recirculating lymphocytes, and the anti-lymphocytic antibodies in ALS are eliminated from the recipients rapidly. Based on our previous studies with ALS in rat vascularized hind-limb transplant, we injected ALS on days −4 and +1, whereby limb grafts were best protected. Since the efficacy of ALS on LEW recipients was different from batch to batch, we examined the absolute number of peripheral lymphocytes 3 days after injection. Only the batches that decreased lymphocyte number by >70% were used in this study. The mean survival time (MST) of vascularized hind-limb transplant in each group was as follows: Group 1: 9.3 ± 1.5 days; Group 2: 44.8 ± 5.5 days; Group 3: 77.0 ± 6.3 days; Group 4: 52.5 ± 5.7 days; Group 5: long-term acceptance (>350 days); and Group 6: 70.8 ± 5.0. Two doses of ALS at days −4 and +1 were insufficient to induce long-term acceptance since ALS monotherapy could hardly prolong the vascularized hind-limb transplant survival. However, ALS was indispensable for vascularized hind-limb transplant tolerance induction as shown in Figure 1. Although CsA could suppress the rejection throughout the therapy, allograft necrosis due to rejection occurred shortly after the immunosuppressant was withdrawn (Figure 1).

### 3.2. Bone Marrow in the Vascularized Hind-Limb Transplant Promotes Tolerance with a Short-Term Low-Level Peripheral Multilineage Chimerism

**Figure 2:**
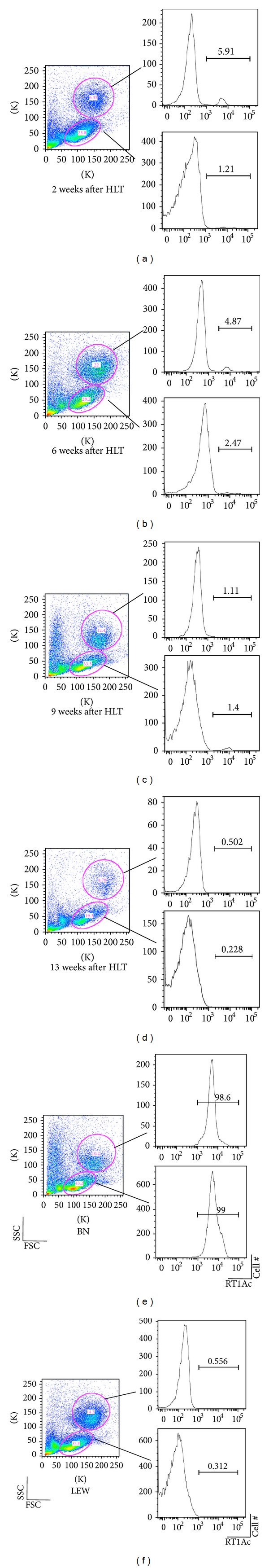
Peripheral blood multilineage chimerism in Group 5. Multilineage peripheral blood persisted for 12-13 weeks following vascularized hind-limb transplant, carried out by hind-limb transplant (HLT), at a low level in Group 5. At 13 weeks following vascularized hind-limb transplant, peripheral blood chimerism could hardly be detected by flow cytometry in 5 of 6 tolerant recipients in Group 5.

Hind-limb is composed of skin, skeletal muscles, bone and bone marrow, and other soft tissues. Bone marrow cells, especially the stem cells, of donor origin are believed to give rise to peripheral hemocytic chimerism, which in turn promotes allograft tolerance. ALS + 45-day CsA treatment induced vascularized hind-limb transplant tolerance (Group 5) while failed in the case of vascularized skin/muscle transplant with MST of 70.8 ± 5.0 days (Group 6). Peripheral blood multilineage chimerism persisted for 12-13 weeks at a low level in Group 5 (Figure 2). At 13 weeks after vascularized hind-limb transplant, we could hardly detect peripheral blood multilineage chimerism by flow cytometry in 5 of 6 tolerant recipients in Group 5. At this point, we employed PCR to detect peripheral blood microchimerism. It turned out that even microchimerism had evanesced (data not shown). The only tolerant recipient with detectable peripheral blood chimerism at 13 weeks after transplant was completely dissociated from peripheral blood chimerism at 18 weeks after transplant (data not shown). After vascularized hind-limb transplantation, there were no clinical signs of GVHD in all groups until the end of this study.

### 3.3. Duration of Immunosuppressive Therapy Plays a Critical Role in Vascularized Hind-Limb Transplant Tolerance Induction

CsA has been widely used in transplantation medicine. It binds to cytoplasmic cyclophilin. Resulting complexes inactivate calcineurin, a crucial enzyme in T-cell receptor signalling. Calcineurin inhibition suppresses interleukin-2 (IL-2) gene transcription and thus inhibits IL-2 production of T cell. In this study, no clinical rejection turned up during CsA treatment. However, if CsA was administered from day 0 to 20, vascularized hind-limb allograft was rejected shortly after CsA was withdrawn, irrespective of ALS (Figure 1). In contrast, 45-day CsA + ALS induced long-term acceptance of the vascularized hind-limb allograft.

### 3.4. Vascularized Hind-Limb Transplant Tolerance Induces Recipient Tolerance to a Secondary Skin and Heart Transplant from the Same Donor Strain

To test the tolerance specificity, a secondary full-thickness skin transplant from both BN and ACI strains was performed when the vascularized hind-limb transplant survived >150 days. All BN skin grafts (*n* = 6) survived indefinitely while all ACI skin grafts (*n* = 6) were acutely rejected 10.0 ± 0.6 days after transplant, which proved the immunocompetence of the tolerant LEW recipients in Group 5.

**Figure 3:**
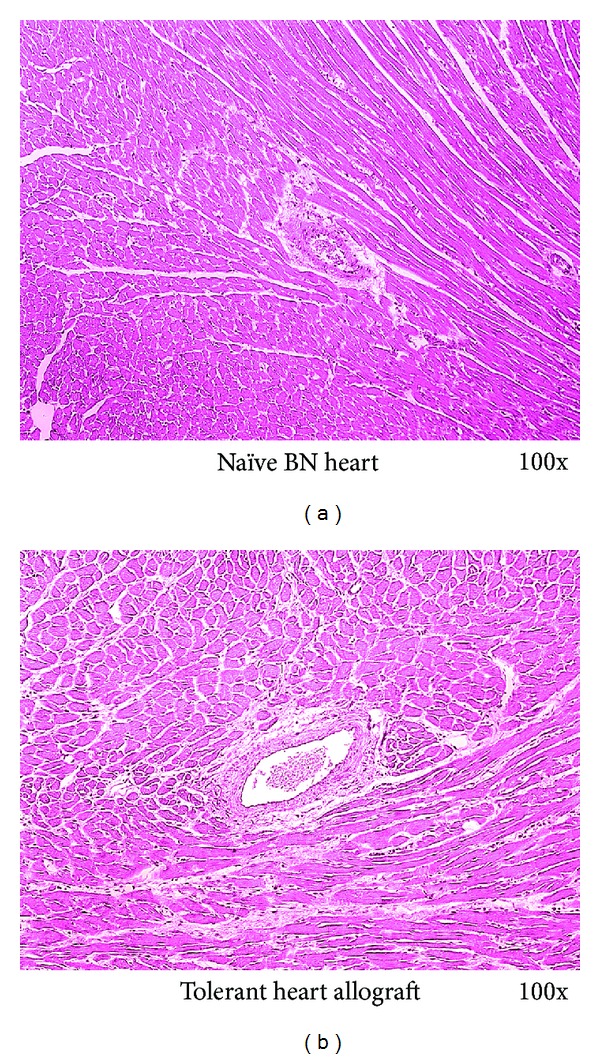
Histology of the secondary heart allograft. Secondary heart transplant from the same donor strain to tolerant LEW recipients in Group 5 was performed after the vascularized hind-limb transplant survived >200 days. Secondary heart allografts showed minimum cellular infiltrate and no haemorrhage, myocardial damage, or signs of cardiac allograft vasculopathy (CAV) >150 days following transplant. 100x.

To test the principle that vascularized hind-limb transplantation is capable of inducing tolerance to solid organs such as the heart from the same donor strain, we performed heart transplant from BN strain to tolerant LEW recipients in Group 5 when the vascularized hind-limb transplant survived >200 days. All heart allografts presented good function with minimum cellular infiltrate and no haemorrhage, edema, myocardial damage or signs of cardiac allograft vasculopathy (CAV) >150 days following transplant (Figure 3).

### 3.5. Clonal Deletion Is a Potential Mechanism of Tolerance Maintenance in This Model

**Figure 4:**
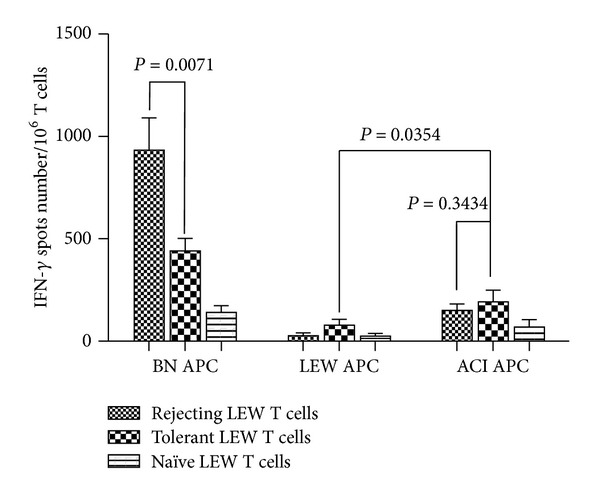
ELISPOT showed a significantly reduced frequency of BN-reactive IFN-*γ* secreting T cells in the spleen of tolerized LEW recipients, compared with that of rejecting LEW recipients (933.3 ± 90.76 versus 440.1 ± 35.17, *n* = 3, *P* = 0.0071). In contrast, rejecting and tolerant LEW had similar frequency of ACI-reactive splenic T cells (151.1 ± 17.78 versus 191.1 ± 32.74, *n* = 3, *P* = 0.3434).

To investigate the possible mechanisms by which tolerance to vascularized hind-limb, secondary skin, and heart allograft was achieved, we performed ELISPOT to detect clonal deletion. ELISPOT is a highly sensitive assay for detecting frequency of cytokine secreting cells, which are IFN-gamma secreting T cells in this study. The ELISPOT showed a significantly reduced frequency of donor-reactive T cells in the spleen of tolerized LEW recipients, compared with that of sensitized LEW recipients and naïve LEW rats as shown in Figure 4.

## 4. Discussion

Heart allograft rejection and lifelong nonspecific immunosuppression used to restrain the alloreaction remain the major obstacle to long-term survival subsequent to clinical heart transplantation [2]. Thus, donor-specific tolerance is deemed as the “holy grail” in the field of transplant. To our knowledge, we are the first to report successful heart graft tolerance induced and maintained by vascularized hind-limb transplant in a fully MHC mismatched rat model (BN to LEW). Tolerance induced in this study is donor specific and is associated with significantly decreased antidonor alloreactions. The most important difference between the current study and others' is that we achieved transplant tolerance without myeloid ablation in a more stringent and clinically relevant model.

Perisurgical lymphocyte ablation is thought to diminish the donor-reactive lymphocyte clones [19]. The fact that absence of ALS abolished tolerance induction, even though CsA was used 45 days after transplant, suggests alloreactive lymphocyte reductive conditioning is crucial for achieving the tolerant state in this experimental model. However, the diminished lymphocyte clone level needs to be maintained for a sufficient period of time by CsA. If no CsA or 21-day CsA was used in the current model, acute rejections displayed shortly after CsA withdraw, implying a pivotal role of this prolonged time window (21-day versus 45-day) for the recipients to be tolerized. Evidence emerged recently that peripheral plasmacytoid dendritic cells are able to contribute to immune tolerance through CCR9-dependent transport of peripheral antigens to the thymus and subsequent deletion of antigen-reactive thymocytes. However, this thymic clonal deletion may be prevented by infectious signals (toll-like receptor signals) [20]. It has also been demonstrated that donor-derived dendritic cells contribute to the central deletion of donor-reactive thymocytes in the recipient thymus [8, 21]. Our ELISPOT assay results are consistent with this concept. In addition, it has been well established that both early and late inflammations promote allograft rejection [22–25]. Thus, a 45-day CsA therapy may have provided sufficient time needed for inflammation caused by transplant surgery to dissolve and central deletion to happen in this study.

In conclusion, we established a novel method for inducing and maintaining heart allograft tolerance in the rat. The findings in this study may open new perspectives for the role of vascularized hind-limb transplant, especially vascularised bone marrow transplant, in the induction and maintenance of organ transplantation tolerance.
